# Emotion Regulation Tendencies and Leadership Performance: An Examination of Cognitive and Behavioral Regulation Strategies

**DOI:** 10.3389/fpsyg.2019.01486

**Published:** 2019-07-02

**Authors:** Brett S. Torrence, Shane Connelly

**Affiliations:** Department of Psychology, University of Oklahoma, Norman, OK, United States

**Keywords:** emotion regulation, leadership, cognitive reappraisal, suppression, attentional deployment, situation modification

## Abstract

Emotion regulation is advocated to be an important factor underlying effective leadership given the task demands and interpersonal stressors facing organizational leaders. Despite the recognition of emotion regulation processes in leadership literature, there is a need for additional theorizing and empirical research on the specific cognitive and behavioral strategies utilized by leaders. This effort attempts to address this gap by examining individual tendencies in four emotion regulation strategies, situation modification, attentional deployment, cognitive reappraisal, and suppression, and their association with leadership task performance. Using an undergraduate student sample, this correlational study assessed the relationship between emotion regulation tendencies and performance in emotionally-relevant domains of leadership. Results provide partial support, suggesting that situation modification and cognitive reappraisal are positively related to leadership performance, whereas suppression was found to relate negatively with performance. Emotion regulation strategies were also found to account for variance in leadership performance above and beyond other emotion-related individual differences. Taken together, these findings suggest that certain regulation processes may be more functional for leaders and extend emotion regulation research in the leadership domain. Theoretical and practical implications of this study are discussed.

## Introduction

Emotions are a central feature of workplace experiences and the tasks and interpersonal demands faced by leaders often arise in emotion-laden contexts. In leadership settings, leaders may need to deliberately modify their emotional experiences and expressions to exercise influence over followers (Humphrey, 2012). Additionally, leaders may need to manage their emotions to facilitate performance on day-to-day tasks (Gooty et al., 2014). In fact, many domains requiring effective leadership, including ethical dilemmas, interpersonal conflicts, and organizational crises (Connelly et al., 2014), call for appropriate emotional responses. Given the considerable influence that emotions have on judgment and behavior (Weiss and Cropanzano, 1996; Kiefer, 2005; Seo and Barrett, 2007), effectively managing emotions is key for successful leadership. Emotion regulation is the “process by which individuals influence which emotions they have, when they have them, and how they experience and express these emotions” (Gross, 1998, p. 275) and, therefore, represents a critical competence contributing to leader performance (Haver et al., 2013).

The study of emotion regulation in leadership has developed alongside the growing research on leader emotion (e.g., Ashkanasy and Tse, 2000). The idea that leaders must display and use emotions to influence leader and follower outcomes has led to the study of concepts, such as emotional labor (Ashkanasy and Humphrey, 2011) and emotional intelligence (George, 2000), in the leadership domain. The emotional labor perspective contends that leaders use tactics such as deep acting and surface acting to control their emotional displays to followers (Gardner et al., 2009) with deep acting being a more effective strategy (Humphrey et al., 2008). Similarly, studies on leader emotional intelligence argue that emotion management is critical for effective leadership as leaders often need to manage their own emotions under the stressful demands of the position (George, 2000). Despite the recognition that emotion management is a part of leadership, our understanding of emotion regulation in leadership is still underdeveloped, particularly regarding explicit emotion regulation strategies.

Unfortunately, sparse empirical work has examined specific cognitive and behavioral regulation strategies used by leaders to manage their own emotions (Gooty et al., 2010). This is unfortunate given that the process by which individuals deal with their emotion influences job performance (Wallace et al., 2009) and leader-follower relationships (Glasø and Einarsen, 2008). Fortunately, recent work on emotion regulation (e.g., Diefendorff et al., 2008; Lawrence et al., 2011) suggests that the process model of emotion regulation (Gross, 1998) represents a well-established framework for furthering our understanding of emotion regulation in the workplace and in leaders. Most importantly, this framework outlines specific behavioral and cognitive strategies that are used to manage different aspects of one's emotional experience, such as situations and thoughts (Webb et al., 2012).

In addition to understanding the strategies used by leaders, the extent to which leaders rely on specific emotion regulation strategies is also a central question given that individuals display different regulation tendencies and that these tendencies can be adaptive or maladaptive depending on the situation. Specifically, emotion regulation strategies differ in their ability to modify emotion (Gross and John, 2003) and their level of effort (Richards and Gross, 2000). Furthermore, the process by which individuals regulate their emotions is not always deliberate (Koole, 2009), suggesting that emotion regulation tendencies may be influential in the day-to-day activities of leaders. As such, there is a need to understand the association between emotion regulation tendencies and leadership effectiveness as research outside the leadership domain shows that regulation tendencies are influential (e.g., Wallace et al., 2009; Liu et al., 2010). Therefore, the purpose of this study is to examine how individual differences in behavioral and cognitive emotion regulation strategies relate to leadership performance.

## Process Model of Emotion Regulation

Emotion regulation is the process responsible for the initiation, maintenance, modification, and redirection of emotional states (Gross, 2008; Koole, 2009; Webb et al., 2012). Gross (2008) describes that emotions emerge from “person-situation transactions” and that the emotion generative process involves four central features: (a) situations, (b) attention, (c) appraisals, and (d) responses. The process model highlights that emotion regulation strategies operate by altering these features of the emotional response (Gross, 1998). Specifically, this model describes that emotions can be regulated through *antecedent-focused* strategies, processes used for modifying the emotional stimulus (e.g., situation) or altering perceptions of the stimulus (e.g., attention), and *response-focused* strategies, processes that alter the emotional response (Gross, 1998). The distinction between antecedent-focused and response-focused strategies lies in the target(s) these strategies regulate. Antecedent-focused strategies address aspects of the emotional response that occur before emotional experiences have become fully activated (e.g., situations, attention, and meaning, Gross, 2002). For instance, individuals may change the situation or direct their attention away from the event to prevent the onset of a negative emotion. Conversely, response-focused strategies are implemented once an emotion and its responses (e.g., experiential, behavioral) are activated (Gross, 2002). An example of a response strategy would be masking outward expressions of anger during a work conflict in order not to appear upset with a co-worker.

Prior research demonstrates that five categories of emotion regulation strategies underlie these two groupings (Gross, 2008). *Situation selection* refers to actions (e.g., approach or avoidance) that increase the likelihood that individuals will be in a situation that produces desirable emotions. *Situation modification* refers to efforts taken by an individual to modify the emotional impact of the situation. *Attentional deployment* involves influencing emotional responses by refocusing attention to different aspects of a situation. *Cognitive change, or reappraisal*, involves altering the emotional meaning of a situation through reappraisal of the emotional stimulus. These four emotion regulation strategies are characterized as antecedent-focused strategies as each of these strategies targets features of an emotional response that occur before an emotion is fully activated. Finally, *response modulation*, or *suppression*, involves inhibiting emotional responses after the emotion has been activated and is a response-focused strategy since its goal is to alter the experiential, behavioral, or physiological response tendencies of an experienced emotion.

Despite the identification of these emotion regulation strategies, the majority of empirical work has focused extensively on two strategies, cognitive reappraisal and suppression. Nonetheless, studies comparing these two emotion regulation strategies provide important findings that cognitive reappraisal, in general, leads to better affective, cognitive, and social outcomes when compared to suppression (Richards and Gross, 2000; Gross and John, 2003; English et al., 2012). However, the central focus on these two strategies has constrained our view of emotion regulation processes and limited our understanding of the different types of responses individuals may employ in emotion-laden settings and their consequences for workplace outcomes (Gooty et al., 2010). Based on the nature of leader performance, employing a broader taxonomy of emotion regulation processes to investigations of leadership is warranted.

## Emotion Regulation and Leadership Performance

The dynamic nature of organizations requires that leaders manage and meet multiple demands and exert influence over their followers. The high degree of complexity and uncertainty characterizing organizational environments has led to the assumption that emotion regulation is necessary for leaders to deal with negative events, establish better leader-member relations, and facilitate job performance (George, 2000; Ashkanasy and Humphrey, 2011; Humphrey, 2012; Haver et al., 2013). An emotion management framework presented by Connelly et al. (2014) identifies several emotion-relevant domains of leadership that require effective emotion regulation, including conflict resolution, inspirational motivation, ethical decision-making, risk taking, and feedback. In these contexts, leaders must think about what emotions are appropriate for the situation at hand and how emotions may drive behavior. Leaders need to remain calm during crises, display enthusiasm and a sense of challenge to garner follower support around a new vision, and portray disappointment when follower performance is lacking, for example.

Even so, emotion regulation is not only needed for altering emotional displays, but for managing the intrinsic impact of emotion on leader decision-making given that emotions influence cognitive processes (Shiv et al., 2005; Seo and Barrett, 2007). Judgment and decision making are fundamental components of leadership (George, 2000) as leaders are often required to formulate and implement decisions that solve social problems in their organization (Mumford et al., 2000). Furthermore, it has been argued that the most pressing situations requiring leader cognition are characterized as ill-defined and uncertain (Mumford et al., 2000) and such situations are likely to elicit emotional responses in leaders. Therefore, the efforts taken by leaders to manage their emotions are critical for ensuring that leaders improve their emotional states and successfully address the problem at hand.

Two central reasons as to how emotion regulation processes impact leadership performance are (a) the relative efficacy of the regulation strategy and (b) the level of effort required to deploy that strategy. First, regulation strategies are differentially effective in their influence of emotional experiences with strategies, such as cognitive reappraisal and perspective-taking, showing better outcomes (Webb et al., 2012). Second, regulation strategies require different resources (e.g., effort and attention) to implement. For instance, expressive suppression is associated with poorer memory compared to reappraisal (Richards and Gross, 2000). Emotion regulation strategies that entail less cognitive effort, in turn reducing off-task attentional pull (Beal et al., 2005), will allow leaders to allocate additional resources toward task performance.

Taken together, emotion regulation tendencies may display varying relationships with leadership performance given that these processes are associated with different consequences. Based on the process model of emotion regulation (Gross, 1998), situation modification, attentional deployment, cognitive reappraisal, and suppression represent regulation strategies of interest to the current effort given that these strategies represent tactics that can be utilized by leaders faced with emotional events on the job. Situation selection was not examined in the present effort based on the notion that leaders may not always have the choice to opt out of, or into, particular situations. Oftentimes, leaders must handle the situation they are dealt, and situation selection represents a strategy that a leader would deploy before they are in a situation, not for managing a specific event. Nonetheless, the behavioral and cognitive processes identified in the current study present an expanded view of emotion regulation in leadership and have, potentially, different implications for performance.

## Situation Modification

Situation modification involves direct efforts taken to change a situation in order to alter its emotional impact (Gross, 2008). This emotion regulation strategy deals with modifying external characteristics of the situation causing the emotion, not managing the emotion itself. For example, if a conflict breaks between two team members during a meeting, a leader may choose to separate the workers or change the topic of conversation to lessen the emotions of the event. Additionally, a leader may interject humor into a stressful situation to lighten the tension of the situation. Unfortunately, there is a lack of research on the effects of situation modification (Gross, 2015). However, this strategy is viewed as a process synonymous to problem-focused coping, which involves managing the problem causing the emotion (Gross, 2002). Research concerning problem-focused coping demonstrates that this strategy is used when situations are viewed as malleable and an individual has control over a situation (Folkman and Moskowitz, 2000). Situation modification, therefore, is likely a viable strategy for leaders given their position of influence and level of control over workplace situations, such as feedback sessions and team settings. Additionally, problem-focused coping is negatively associated with stress symptoms and positively related to adjustment, as well as increases in positive moods (Riley and Park, 2014). These findings suggest that situation modification may represent an adaptive strategy for leaders in managing their emotional response. However, this emotion regulation strategy likely requires that a leader recognize the potential paths of a situation which suggests that a requisite level of emotional knowledge and emotion understanding is needed (Joseph and Newman, 2010; Connelly et al., 2014). For instance, understanding elements that give rise to emotional responses may improve a leader's ability to effectively alter the emotional event. Nonetheless, in general, having the tendency to modify situations in order to alter its emotional impact is likely an effective strategy for leaders. Thus, our first hypothesis:

*Hypothesis 1*: Situation modification is positively related to performance on leadership tasks.

## Attentional Deployment

Attentional deployment refers to the process by which individuals attend to different aspects of a situation to influence emotions (Gross, 2008). Two potential avenues of attentional deployment include distraction and concentration. Distraction involves redirecting attention away from emotional aspects of the situation (Webb et al., 2012). Distraction may include a leader thinking about something positive in order to distract themselves from negative emotions elicited from a feedback session. On the other hand, concentration involves focusing on emotional elements of a situation (Gross, 2008). In the ethical dilemmas, leaders may concentrate on their emotions to think about causes of the situation and their feelings. The concept of concentration is closely tied to rumination, a process which involves focusing on thoughts and feelings associated with an emotion-laden situation (Gross, 2008).

In leadership settings, attentional deployment could serve leaders adaptively if used in a manner that suits situational demands. While meta-analytic work by Webb et al. (2012) found that distraction was effective in changing emotional experiences and concentration was not, in terms of producing positive effects on leadership outcomes both distraction and concentration could lead to beneficial effects. Attentional deployment, in the form of distraction and/or concentration, may allow a leader to operate within emotional settings. When resources are low and on-task attention is needed, attentional deployment can allow a leader to operate within stressful environments without being susceptible to a strong emotional pull. While emotions can serve adaptive functions for decision making processes (Lerner et al., 2015), the effective management of organizational issues requires attending to other components as well. Therefore, we predict the following:

*Hypothesis 2*: Attentional deployment is positively related to performance on leadership tasks.

## Cognitive Reappraisal

Cognitive reappraisal strategies target thoughts surrounding an event by changing the way the situation and/or the emotional demands are perceived (Gross, 2008). Reinterpretation and perspective-taking comprise two core elements of cognitive reappraisal (Webb et al., 2012). For example, when facing a crisis, a leader may modify the meaning of the situation to alter its emotional impact. Empirical research on cognitive reappraisal has found that this strategy is associated with several functional outcomes (Gross, 2013, 2015). Cognitive reappraisal is associated with the experience of more positive emotions and decreased levels of negative emotions (Gross and John, 2003). Additionally, studies on cognitive reappraisal demonstrate improved cognitive functioning (e.g., memory; Richards and Gross, 2000), better social functioning (Gross and John, 2003), and higher task performance (Wallace et al., 2009).

Cognitive reappraisal appears to be a particularly functional emotion regulation strategy for leaders on the basis that this form of regulation targets evaluations and judgments of emotion-laden events (Koole, 2009). Broadening one's perspective on a situation can allow a leader to increase their situational awareness and understanding through reinterpretation and perspective-taking. This process not only serves to moderate leaders' emotional experiences but improves their ability to address the situation at hand. Furthermore, cognitive reappraisal is classified as a goal-oriented strategy indicating that these actions are likely to be aligned with task and interpersonal priorities (Koole, 2009). Thus, we predict the following:

*Hypothesis 3*: Cognitive reappraisal is positively related to performance on leadership tasks.

## Suppression

Suppression, the most common form of response modulation, refers to efforts that inhibit experiences and expressions of emotion (Gross, 2008). Employed after an emotion is generated, suppression targets experiential and/or behavioral responses (Webb et al., 2012). For example, when dealing with an angry subordinate, a leader may choose to maintain a neutral expression and mask their frustration. In terms of effectiveness, suppression is often considered maladaptive given its relationship with adverse outcomes. Suppression is ineffective in decreasing one's experience of emotions (Gross, 1998), negatively impacts social functioning (Butler et al., 2003), requires more effortful processing (Richards and Gross, 2000), and is negatively related to task performance (Wallace et al., 2009).

Similar to reappraisal, suppression has been defined as a goal-oriented strategy, frequently employed to facilitate the attainment of goals. However, instead of targeting knowledge-related responses, suppression focuses on emotional expressions (Koole, 2009). Even though leader emotional expressions can impact follower outcomes (Van Kleef et al., 2009), suppression fails to target other performance elements impacted by emotional responses. Given that suppression does not address emotional targets that have a more direct impact on performance, such as knowledge, suppression is likely to be a poor strategy for leaders. Therefore, our next hypothesis:

*Hypothesis 4*: Suppression is negatively related to performance on leadership tasks.

## Emotion-Related Individual Differences

Emotion regulation tendencies should also account for unique variance in leadership performance beyond other emotion-related individual differences, such as empathy and trait affect. Empathy, the ability to perceive and experience others' emotion, demonstrates positive relationships with perceptions of task and relational leadership (Kellett et al., 2006) and is argued to be indicative of a leader's perspective taking ability (Wolff et al., 2002). Trait affect, or the tendency to experience positive or negative emotions, also displays modest relationships with leadership outcomes (e.g., effectiveness, emergence; Joseph et al., 2015) and, therefore, may explain variance in leader performance. However, given that emotion regulation is argued to have a more direct influence on performance (Joseph and Newman, 2010), we expect emotion regulation strategies to account for variance beyond empathy and trait affect. Thus, our final hypothesis:

*Hypothesis 5*: Emotion regulation tendencies will account for unique variance in performance on leadership tasks above and beyond empathy and trait affect.

## Method

### Sample and Procedures

A total of 226 undergraduates (77% female, 23% male) from a large, southwestern university participated in this study for course credit during Fall 2015. Participants were recruited using a university-based online research website and were required to be 18 years of age or older to participate. On average, participants were 18.8 years old (*SD* = 1.40) and had 2.28 years of work experience. Thirteen percent (*n* = 26) of participants had no working experience, but of those reporting work experience only 5% had <6 months of experience. Participants reported several types of work experience as well with ~56% having worked in service/retail industries, 8% in administrative roles, and 7% in teaching and coaching roles. Thirty participants were dropped from the final analyses for careless responding (i.e., failing bogus items; Meade and Craig, 2012) or failing to complete the relevant survey measures. The final sample consisted of 196 students.

Participants completed the survey using an online-based data collection system. This study was approved by the Institutional Review Board (IRB) for the Protection of Human Subjects at the University of Oklahoma prior to data collection. Participants provided unsigned online consent in accordance with the guidelines established by the IRB at the University of Oklahoma before beginning the study. After providing consent, participants completed a series of self-report measures including a questionnaire of emotion regulation tendencies. Next, participants were asked to take on the role of a leader in hypothetical scenarios and make explicit decisions to three different performance situations. Appendix A presents the leadership scenarios. These scenarios reflected diverse domains of leadership performance identified as relevant to emotional responses and regulation: ethical decision-making, negative feedback, and high-stakes situations (Connelly et al., 2014). After responding to these vignettes, participants completed another series of measures and a demographics questionnaire.

### Measures

#### Emotion Regulation Strategies

The development of emotion regulation scales began with an extensive review of the emotion regulation literature (e.g., Gross, 1998, 2008; Koole, 2009; Lawrence et al., 2011; Webb et al., 2012) as well as a review of existing self-report measures (e.g., Emotion Regulation Questionnaire, Gross and John, 2003). Following this review, the first and second author created or adapted items to represent the four proposed emotion regulation strategies using deductive procedures (Hinkin, 1998). In total, 24 items were written to tap onto the definitions for situation modification, attentional deployment, cognitive reappraisal, and suppression. Following item development, items were reviewed by an expert in emotion regulation research to ensure that each item adequately reflected its intended construct. To assess the factor structure of the emotion regulation measure, a series of confirmatory factor analyses were conducted. Results provide moderate support for the proposed four-factor model; however, four items with significant cross-loadings were removed. The four-factor solution provided mediocre fit (χ^2^ = 354.89, *df* = 146, CFI = 0.87, RMSEA = 0.09, SRMR = 0.08) and the standardized factor loading for all items exceeded 0.40. The theoretical model provided better fit than a single-factor model (χ^2^ = 797.99, *df* = 152, CFI = 0.59, RMSEA = 0.15, SRMR = 0.14) and two-factor model (χ^2^ = 512.35, *df* = 151, CFI = 0.77, RMSEA = 0.11, SRMR = 0.14). However, the four-factor model fit as well as the three-factor model (χ^2^ = 363.41, *df* = 149, CFI = 0.87, RMSEA = 0.09, SRMR = 0.08). The four-factor solution was retained for conceptual and theoretical clarity. These measures are described in the subsequent paragraph.

*Situation modification*, or the extent to which an individual engages in direct action to modify an emotional situation (Gross, 2015), was assessed with three items (i.e., “When I want to feel more positive emotion, I can change aspects of the situation in order to do that,” “I can change the emotional nature of a situation by injecting humor into the situation,” and “I manage my emotions by changing aspects of the situation”; α = 0.61). *Attentional deployment*, or the extent to which an individual redirects or shifts their attention in emotional situations, was assessed via four items (i.e., “I am able to distract myself from strong emotions,” “When I am in an emotional situation, I manage my emotions by focusing on non-emotional aspects of the situation,” “It is hard for me to stop thinking about the emotions I am feelings” (reverse-scored), “When experiencing negative emotions, I am unable to think about anything but that emotion” (reverse-scored); α = 0.73). *Cognitive reappraisal*, or the extent to which one changes their appraisal of an emotional situation (Gross and John, 2003), was assessed via seven items (i.e., “When I want to feel more positive emotions, I can change my perspective on the situation,” “When I want to feel less negative emotions, I can change my perspective on the situation,” “I can control my negative emotions by changing the way I think about the situation,” “I can control my positive emotions by changing the way I think about the situation,” “I manage my emotions by changing my perspective on the situation I am in,” “I can increase my feelings of positive emotions by thinking about different aspects of the situation,” “I can decrease my feelings of negative emotions by thinking about different aspects of the situation”; α = 0.88). Items for this scale were drawn or adapted from the ERQ (Gross and John, 2003). *Suppression*, or the extent to which attempts to inhibit their emotional expressions, was assessed via five items (i.e., “I generally try not to show my negative emotions,” “I keep my emotions to myself,” “I manage my emotions by not expressing them,” “I do not express my negative emotions,” “I generally try not to show my positive emotions”; α = 0.78). Items for this scale were also drawn or adapted from the ERQ (Gross and John, 2003). All scales were rated using a 7-point Likert scale (1 = *strongly disagree*, 7 = *strongly agree*).

#### Performance

Between-person differences in leadership task performance were assessed using constructed scenarios. Participants responded to three written vignettes describing an ethical dilemma, negative feedback, and a high-stakes situation, respectively (see Appendix A). These domains of leadership were selected based on prior research suggesting that these domains represent emotionally-relevant tasks performed by leaders (Kligyte et al., 2013; Connelly et al., 2014; Johnson and Connelly, 2014). Given that a student sample was used in the current study, vignettes were developed to match the knowledge and abilities of an undergraduate sample (Wason et al., 2002; Aguinis and Bradley, 2014). To enhance scenario realism and improve the generalizability of the study, the situations were ground in leadership contexts familiar with students (i.e., on-campus groups, service industry).

Given the applicability of vignettes for assessing decisions and judgments (Aguinis and Bradley, 2014), performance on the constructed scenarios was evaluated in terms of social judgment skills, a key determinant of leadership performance (Connelly et al., 2000; Mumford et al., 2000). Social judgement represents a leader's ability to make a decision that is workable within the goals, demands, and constraints of the social setting (Mumford et al., 2000), therefore, responses were rated on quality (*r*${}_{\text{wg}}^{*}$ = 0.85), considering others' perspective (*r*${}_{\text{wg}}^{*}$ = 0.80), social perceptiveness (*r*${}_{\text{wg}}^{*}$ = 0.84), and good judgment under uncertainty (*r*${}_{\text{wg}}^{*}$ = 0.81). Trained raters coded participant responses on these dimensions. Raters received frame-of-reference training (Bernardin and Buckley, 1981) and were provided benchmark ratings scales that reflected high, medium, and low levels of each dimension. Construct definitions and benchmark responses are shown in Appendix B. A general factor accounted for 98% of the variance in the leadership outcome variables, so scores were aggregated across scenarios to create a composite performance score (*r*${}_{\text{wg}}^{*}$ = 0.83).

#### Covariates

Covariates were included to account for individual differences that may influence leadership performance. The Positive and Negative Affect Schedule—Modified (Hepler and Albarracin, 2013) measured positive (19-item; α = 0.93) and negative (19-items; α = 0.82) trait affectivity on 7-point scale (1 = *very slightly or not at all*; 7 = *extremely*). Empathy (α = 0.89) was measured via 16-items on a 5-point scale (0 = *never*; 4 = *always*) using the Toronto Empathy Questionnaire (Spreng et al., 2009). Demographics, including gender, were also measured given potential gender differences in leadership (Eagly et al., 2003).

## Results

### Hypothesis Testing

Table 1 displays the means, standard deviations, correlations, and reliabilities for all variables. As shown in Table 1, situation modification, cognitive reappraisal, and suppression demonstrate correlations with performance in the expected direction. Additionally, regression analyses were conducted to assess the relationship between emotion regulation tendencies and performance on leadership tasks. In support of Hypothesis 1, situation modification was found to be positively associated with performance (β = 0.14, *t*_(194)_ = 2.04, *p* = 0.04). Attentional deployment was unrelated to performance (β = −0.03, *t*_(194)_ = −0.44, *p* = 0.66), providing no support for Hypothesis 2. Cognitive reappraisal displayed a positive relationship with performance (β = 0.19, *t*_(194)_ = 2.76, *p* = 0.006) supporting Hypothesis 3. Finally, in support of Hypothesis 4, suppression was found to be negatively related to performance (β = −0.18, *t*_(194)_ = −2.56, *p* = 0.01). These results support the overarching idea that habitual tendencies in emotion regulation strategies play a role in leadership performance. Specifically, situation modification and cognitive reappraisal appear to be beneficial emotion regulation strategies given their positive association with performance, whereas suppression appears to be harmful as a tendency to suppress emotions and was negatively related to performance.

**Table 1:** Descriptive statistics and correlations of emotion regulation, performance, and covariate measures.

	***M***	***SD***	**1**	**2**	**3**	**4**	**5**	**6**	**7**	**8**	**9**
1. Gender	0.78	0.42	−−								
2. SM	4.71	1.04	−0.12	(0.61)							
3. AD	3.50	1.11	−0.14^*^	0.43^**^	(0.73)						
4. CR	4.94	1.03	0.04	0.67^**^	0.38^**^	(0.89)					
5. Suppression	3.78	1.23	−0.16^*^	−0.07	0.32^**^	−0.13	(0.78)				
6. Empathy	3.99	0.53	0.33^**^	0.15^*^	−0.10	0.26^**^	−0.25^**^	(0.89)			
7. Positive affect	4.73	0.88	−0.07	0.43^**^	0.28^**^	0.46^**^	−0.21^**^	0.25^**^	(0.93)		
8. Negative affect	3.04	0.91	0.09	−0.33^**^	−0.41^**^	−0.39^**^	−0.10	−0.09	−0.53^**^	(0.82)	
9. Performance	2.99	0.49	0.15^*^	0.14^*^	−0.03	0.19^**^	−0.18^*^	0.19^**^	0.03	−0.14^*^	(0.83)

*N = 196*.

* *p < 0.05*.

** *p < 0.01. For gender, 0 = male, 1 = female*.

*SM, situation modification; AD, attentional deployment; CR, cognitive reappraisal. Reliabilities are presented along the diagonals*.

Next, hierarchical regression analyses were performed regressing performance on situation modification, cognitive reappraisal, and suppression controlling for gender, trait affect, and empathy. Attentional deployment was excluded from these analyses given its null relationship with performance. Table 2 displays results from the hierarchical regression analyses. As shown in Table 2, negative affect and empathy were significantly related to performance on the leadership tasks. Specifically, higher levels of negative affect were associated with lower performance scores and higher levels of empathy were associated with higher performance scores. These findings indicate that a propensity to experience negative affect was associated with decreased performance, whereas individuals higher in empathy displayed better performance across these tasks. Next, the inclusion of situation modification, cognitive reappraisal, and suppression in the model explained unique, incremental variance above the covariates. Suppression was a significant predictor of leader performance, whereas situation modification and cognitive reappraisal were not significant predictors of performance above and beyond the other variables in the model. These results suggest that individuals who tend to suppress their emotions may perform less effectively in emotionally-relevant leadership domains. Furthermore, a potential explanation for the lack of incremental variance accounted for by situation modification and cognitive reappraisal with performance, respectively, is the strong correlation (*r* = 0.67) between the two emotion regulation strategies. As such, partial support was found for Hypothesis 5 as suppression was the only emotion regulation strategy to relate to performance above and beyond the emotion-related traits of positive affect, negative affect, and empathy.

**Table 2:** Hierarchical regression analysis of leadership performance on emotion regulation strategies.

**Variables**	**Model 1**	**Model 2**
Gender	0.10	0.10
PA	−0.11	−0.20^*^
NA	−0.20^*^	−0.16
Empathy	0.16^*^	0.11
Situation modification		0.10
Cognitive reappraisal		0.10
Suppression		−0.15^*^
*F*	3.71^**^	3.48^**^
Δ*F*		3.02^*^
*R^2^*	0.07^**^	0.11^**^
Δ*R^2^*		0.04^*^

*N = 196*.

* *p < 0.05*.

** *p < 0.01. Standardized beta coefficients are presented*.

## Discussion

Leaders deal with a variety of emotion-laden events in their day-to-day workplace activities. From managing conflict among followers to planning under conditions of crisis, leaders frequently perform in situations that give rise to emotional experiences. Responses to these events are critical for performance based on research suggesting that certain emotion regulation strategies are more adaptive than others. Given that our understanding of specific strategies for regulating emotions in leadership contexts is underdeveloped, this study sought to explore the relationship between emotion regulation tendencies and performance on leadership tasks. Building on recent work on emotion regulation in the workplace (Lawrence et al., 2011), we investigated specific strategies that leaders may rely on once in an emotion-eliciting event: situation modification, attentional deployment, cognitive reappraisal, and suppression. Compared to other emotion regulation perspectives, the process model of emotion regulation (Gross, 1998) provides a range of specific emotion regulation strategies for managing different aspects of emotion-laden events. Specifically, this model highlights different strategies that address different targets, namely situation, attention, appraisals, and expressions, surrounding an emotional response. This perspective better captures the range of actions leaders use to deal with their emotions, an area currently lacking in leadership research (Gooty et al., 2010).

Findings from the current study suggest that there is a positive relationship between situation modification, cognitive reappraisal, and performance on leadership tasks, respectively. On the other hand, individuals reporting higher levels of suppression performed worse on these tasks. Interestingly, attentional deployment was found to be unrelated to performance. Taken together, these results extend common findings in the emotion regulation domain to the leadership context. Specifically, preliminary evidence from this study suggests that there may be a benefit for leaders in using situation modification and/or cognitive reappraisal when managing emotional workplace demands. Leaders with a preference modifying emotional situations are likely to possess the knowledge needed to understand how emotions operate and have the skills necessary to change the emotions in the present context making them effective emotion managers. Similarly, cognitive reappraisal, which alters emotion through reinterpretation and perspective-taking, is a beneficial strategy for leaders as engaging in reappraisal may facilitate processes that improve a leader's understanding of the situation as a whole, its meaning, and other people's (e.g., followers) perspective. This finding is supported by a long line of research assessing habitual use of cognitive reappraisal (Gross, 2013).

In contrast, a tendency to suppress emotional experiences may hinder performance in emotion-laden events. In terms of habitual use, suppression has long demonstrated detrimental effects with various criteria of interest (Gross, 2015). Even though suppression is effective for masking emotional responses, this process fails to address emotional elements directly underlying leader judgment and performance. As opposed to cognitive reappraisal which targets knowledge, suppression solely addresses responses (Koole, 2009). While potentially useful in certain interpersonal contexts, leaders who continually suppress their emotions are likely to be less effective and may experience higher levels of stress and burnout (Brotheridge and Grandey, 2002). Lastly, attentional deployment did not help or hurt performance on these leadership tasks. To better understand the role of attentional deployment strategies in leadership performance, future research should assess the different dimensions of attentional deployment, distraction, and concentration (Webb et al., 2012), as these strategies may have differential effects for leaders (Little et al., 2012).

## Implications

From a leadership perspective, this effort provides empirical support to prior conceptual work on the role of emotion regulation in leadership and organizations (Riggio and Reichard, 2008; Ashkanasy and Humphrey, 2011; Lawrence et al., 2011). A predominant assumption is that emotion management is a necessary component of effective leadership. However, results from this study indicate that certain emotion regulation strategies may be more advantageous than others. Specifically, situation modification and cognitive reappraisal appear to be to functional strategies for managing emotion-laden events in the workplace. Furthermore, given that emotion regulation responses can occur implicitly (Gross, 2008), identifying the influence of individual differences in emotion regulation on leadership outcomes seems appropriate. Leaders with a tendency to employ adaptive regulation strategies are likely to be better suited for handling the task and interpersonal demands associated with leadership positions.

Furthermore, findings from this study lend support to the use of the Gross (1998) process-model of emotion regulation in leadership settings. The vast majority of research on emotion management in leadership has investigated this construct from the perspective of emotional labor (Humphrey, 2012) and emotional intelligence (Kerr et al., 2006). Even though these approaches have provided supporting evidence for the role of emotion regulation at work, the broader set of strategies outlined in this model provides a more fine-grained picture of the emotion regulation process. As opposed to simply managing their emotional expressions, leaders may control their emotions be modifying situations, changing their thoughts, or diverting their attention. Given that situations, situational elements, meanings, and expressions all represent different components of emotion-laden events, incorporating theory that encompasses these aspects into our understanding of emotion regulation in leadership is warranted.

From a practical perspective, understanding individual differences in emotion regulation appears to be important for informing training interventions aimed at improving leader emotion regulation capabilities. Connelly et al. (2014) discuss that getting leaders to recognize the regulation strategies they rely on as well as the relative effectiveness of these strategies is critical for improving emotion regulation usage. By recognizing their emotion regulation tendencies, leaders can focus on improving and building upon their effective strategies in addition to developing alternative emotion management approaches. For instance, helping leaders who are prone to suppression understand the detrimental effects of this strategy and assisting them in developing alternative regulation strategies may be more effective than simply training them on the different types of regulation strategies (Connelly et al., 2014).

## Limitations and Future Research

Several limitations of this study should be noted despite its potential implications. First, while the pattern of results uncovered here does align with prior research on emotion regulation, the results of the present effort should be taken with caution given measurement issues of the scale. Specifically, the situational modification scale exhibited low reliability (α = 0.61) indicating low internal consistency and modest levels of variance attributable to error. The use of three items for assessing situation modification likely contributes to the low reliability as well. Further measure development is needed to correct these issues. Alternatively, the low reliability of this scale suggests that individuals may exhibit more variability in their use of situation selection strategies. Another limitation is the strong correlations displayed between the cognitive reappraisal, situation modification, and attentional deployment scales. This issue suggests that responses on these scales may influenced by standing on a higher-order factor (e.g., antecedent-focused strategies) or that the items assessing these constructs were not distinct enough. The Gross (1998) model of emotion regulation categorizes situation modification, cognitive reappraisal, and attentional deployment under the dimension of antecedent-focused strategies, as such, positive correlations between these strategies is not unexpected. However, given this study's interest, the level of abstraction was kept at the specific families of emotion regulation strategies. Furthermore, the measurement model for the four-factor emotion regulation measure displayed mediocre fit compared to the recommended cutoff scores (Hu and Bentler, 1999). However, Chen et al. (2008) show that small sample sizes (*n* < 200) have an influence on model fit estimates. Future work will need to cross-validate this four-factor model using a larger sample.

Another limitation is that this study was conducted with an undergraduate sample limiting its generalizability to a leadership sample. A sample with more leadership experience may demonstrate better performance in feedback, ethical decision-making, and high-stakes situations. However, the low-fidelity performance tasks were ground in leadership situations familiar to undergraduate students. Furthermore, a more experienced leadership sample may display differences in emotion regulation given that emotion regulation tendencies vary over one's lifetime. Although the developmental nature of emotion regulation suggests that strategy usage varies with age (Gross, 2013), the relationships found with these strategies are consistent with prior research and appear to hold across age groups (Nolen-Hoeksema and Aldao, 2011). Also, the paper-and-pencil nature of the vignettes is another potential limitation. The use of a low-fidelity simulation may not accurately capture the emotionally-laden nature of real-world workplace situations. While this method allowed for the assessment of decisions across different situations, participants responses may differ when facing these issues in actual organizational settings. Another limitation may be the manner in which leadership performance was evaluated. Participant decisions were rated on quality, considering others' perspective, social perceptiveness, and good judgment under uncertainty. However, the process of considering another person's perspective or being socially perceptive may be intertwined with cognitive reappraisal as it requires the ability to consider alternative perspectives of a situation, for example. Nonetheless, effective leadership calls for the development of decisions that work within the interpersonal and organizational setting (Mumford et al., 2000) and emotion regulation strategies, such as cognitive reappraisal, may serve to inform leader decisions by facilitating these processes. Still, additional research assessing emotion regulation strategies with other leader performance outcomes is needed.

This study was concerned with emotion regulation tendencies and its relationship with leadership performance. However, certain individual differences may also contribute to a leader's ability to regulate emotion. Emotion recognition, the ability to identify emotions, may be a precursor to effective emotion regulation (Joseph and Newman, 2010). On the other hand, personality constructs such as alexithymia, which reflects the inability to identify and describe emotions (Lennartsson et al., 2017), are likely to have detrimental effects on a leader's ability to properly regulate their emotions in the workplace. Future research should examine individual differences that benefit and hinder leader emotion regulation (Gooty et al., 2010). Additionally, the effectiveness of strategies may depend on context. While research demonstrates that certain emotion regulation strategies are more habitually functional (Aldao et al., 2010), that does not negate the idea that maladaptive strategies may be functional in the right settings. Future studies on leader emotion regulation should assess the role contextual factors and emotion regulation choice (Sheppes et al., 2014) on interpersonal and performance outcomes. Stress, uncertainty, and crisis contexts represent different organizational events that leaders must face, and the emotion regulation strategy leaders choose to use likely influences their ability to successfully deal with the event.

Finally, this study looked at the individual effect of each strategy on performance. However, leaders are likely to use emotion regulation strategies conjointly or rely on multiple regulation strategies to alter their emotional experiences. Recent work on emotional labor by Gabriel et al. (2015) suggests that individuals exhibit different profiles of emotional labor use and shows that these profiles exhibit differential outcomes on exhaustion, satisfaction, and authenticity. Given that leaders are likely to utilize different emotion regulation strategies throughout their worklife, investigating the influence of emotion regulation patterns effects on leadership outcomes such as well-being, leader-follower relationships, and performance is worthy of future research.

## Conclusion

In this study, we investigated the relationship between individual differences in emotion regulation strategies and performance on leadership tasks. Incorporating strategies from the Gross (1998) process-model of emotion regulation, we examined four strategies that leaders may use once they find themselves in an emotion-laden event: situation modification, attentional deployment, cognitive reappraisal, and suppression. The effectiveness of these strategies was determined by performance on leadership tasks. Results from this effort demonstrate that situation modification and cognitive reappraisal are positively associated with performance, suppression is negatively related to performance, and attentional deployment has no relationship. Furthermore, suppression accounted for performance in leadership tasks above and beyond gender, trait affectivity, and empathy. From a practical standpoint, these results suggest that the certain emotion regulation strategies may be more functional for leaders and the emotion regulation strategy relied on by a leader may facilitate or hinder their effectiveness.

## Ethics Statement

This study was carried out in accordance with the recommendations of the University of Oklahoma Institutional Review Board with informed consent from all subjects.

## Author Contributions

All authors listed made substantial, intellectual contributions to the work, and approved it for publication.

### Conflict of Interest Statement

The authors declare that the research was conducted in the absence of any commercial or financial relationships that could be construed as a potential conflict of interest.
