# Supplementary material for: Emotion Regulation Tendencies and Leadership Performance: An Examination of Cognitive and Behavioral Regulation Strategies
Source: Front Psychol. 2019 Jul 2;10:1486. doi: 10.3389/fpsyg.2019.01486 (PMC6614202; doi:10.3389/fpsyg.2019.01486)
Supplement: Supplementary file 1 [file Data_Sheet_1.pdf]

Appendix A: Leadership Performance Scenarios

Ethical Decision-Making

You are in a leadership role in a sorority/fraternity and are in charge of organizing a large fundraising charity event. This event is highly visible one in the community and will reflect on the local and national chapters of your sorority/fraternity, so things must go smoothly. You are managing and coordinating the efforts of more than a dozen people who are helping with various aspects of the event. One of the volunteers pulls you aside and tells you that he suspects that another volunteer, the treasurer and a personal friend of yours, has been misusing funds that were collected for the event. You do some investigating and find something is wrong with the financial accounting, but you don't have any proof of wrongdoing. You are worried that word of some wrongdoing will get out and negatively impact this event. How will you address this situation? What will you say to your friend?

Negative Feedback

You are a shift manager at a food service company. Low sales have caused corporate to enact new company policies nationwide. Since you are a shift manager, it is your duty to enforce these new policies. However, due to the nature of the new policies, not all employees have been welcoming to the changes. Specifically, you have noticed that one of your more senior and better performing employees has been exhibiting noticeably less effort and encouraging others to do the same. Further, you have heard rumors that this employee has been bad mouthing you behind your back. The rumors have not only attacked your character, but have undermined your skills and abilities as a manager. Your company cannot remain functional if this type of behavior exists, but you know this person has shown promise in the past. What are you going to do? Provide details about what you will do and provide a rationale for your actions.

High-stakes Situation

You are in a leadership role in an on-campus student group. You hold weekly meetings with other elected members to discuss various issues, such as ways to increase visibility within the community, increase student involvement, and future plans for your group. Over the year, you have gotten to know all the other members well and have developed strong friendships within the group. You have begun to notice that one of your more energetic group members has been less talkative and enthusiastic. You do not give it much thought and attribute the behavior to her just having a bad day. However, as weeks go by, you notice that her behavior has not changed and in fact has gotten worse. After your most recent meeting, other group members approach you with details about the member’s unusual behavior. They explain that her boyfriend has been verbally abusive and controlling and she feels as though she is stuck in this situation. Everyone is turning to you for help and guidance in this situation. How will you approach this situation and what are you going to do? Provide details about what you will do and provide a rationale for your actions.

Appendix B: Benchmarks for Evaluating Performance

Quality

Definition: The degree to which the solution is realistic, practical, and appropriate for the situation.

- Completeness: Did the participant understand the issue/situation at hand? Was the relevant information addressed?
- Coherence: Was the response well thought out and logical?
- Usefulness: Is the solution a realistic and appropriate response to the situation at hand?

Example anchors.

1	2	3	4	5
I am going to try and fix the situation in the best way possible.	I would probably fire the person. With people that are against the management there will not be the best teamwork going on therefore they shouldn't be working there anymore.	I would talk to this personally and express my concern and feelings. I would tell them how important they are to the company and how much I admire them for their hard work.	I would hold a meeting with all of the employees of the company. In the meeting I would bring up the recent changes and ask the employees if they would like to speak about these changes. This would allow the employees to have a comfortable environment to confront the problems they see with the changes.	I would let them know that I too, feel that some of the policies are not ideal but seeing as though we are employees, we must obey the policies or risk the loss of our jobs. I would let the employee know that I am there for them and hope that we can both move on from this and go with the flow of the new company policies even though we don't particularly care for it.

Considering Others' Perspective

Definition: The degree to which multiple perspectives were considered in making the decision.

- Were others' potential reactions to the solution considered?
- Were multiple points of view taken into consideration?

Example anchors.

1	2	3	4	5
I would simply ask my friend if they knew anything about the money. If they are truly my friend, they would tell the truth.	I would tell my friend that I will confront him. I will then go confront the treasurer and ask him/her if they did it or not and that they should be honest with me.	I would personally go and talk to the treasurer and see what is going on. I would maybe look at recent receipts and any kind of paper work that would show any recent purchases, and maybe that way I could figure out what the problem was. If it looks like that person has stolen or misused their privileges, then I would go to the higher authority members and have them help me handle the situation.	I would first tell the volunteer to keep this information between us because we don't want to cause worry about a rumor that has no proof. I would tell my friend that I need to discuss something with them privately. I would bring proof of the incorrect financial accounting to the private meeting and just ask what was going on. I wouldn't accuse the friend of anything since I don't have any proof.	First off, I would address my friend about the financial problem. I would not blame her, but simply state that some things are off and ask if she noticed anyone else using the funds or maybe stealing from the group. This takes the personal blame off of her and will allow her to admit to something being not right without saying that it is her fault.

Social Perceptiveness

Definition*:* The degree to which the response involved awareness of the needs, goals, and demands of others and relationships with other.

- Did the participant describe how others could influence the situation?
- Did the participant attempt to address the goals and needs of others?
- Does the participant appear to be aware of the system/social breakdown?

Example anchors.

1	2	3	4	5
I would first confront this person about the problem at hand and remind them that I have more power over them. I would tell them that it is my duty to enforce these new policies and a failure to follow the rules will result in termination.	I would confront the employee that was talking bad about me behind my back and ask them what their problem was. As a manager if there is a problem, I would prefer that the employee come to me and say these things to my face.	If it is not fixed, I would tell them that their position would be terminated. I would give them specific goals to be met and I would tell them to create a more positive atmosphere in the work place.	As the new policies are out of my control, I will remind the employee that I am simply doing my job by enforcing the new changes, and that I understand their unhappiness with the changes, but that it is also their job to abide by the rules of the company. I will be forced to explain that if the behavior continues, that I will be forced to take action.	I would meet with this employee privately and address that there have been some accusations of negative attitudes. I think the employee needs a safe space where they could express their concern and problems with the policy changes honestly. This employee needs to feel like someone is listening to their opinion and cares about them. I would also tell them that the company cannot function if one employee is encouraging the others to stop performing as well as they can.

Good Judgment Under Uncertainty

Definition: The degree to which the participant is willing to make decisions and take appropriate action in the uncertain situation.

- Does the participant appear willing and able to make a good decision?
- Does the participant recognize the limitations of the situation and the limitations of their knowledge of the situation?
- Was appropriate action taken even though the correct solution may be ambiguous?

Example anchors.

1	2	3	4	5
I would not worry about it. I would say tell no one about this until after the fundraiser. I would confront the man who is stealing and ask if it's true and kick him off the team if it is true.	I would confront the friend directly and ask her if she knows what is going on. I wouldn't accuse her, but I would talk to her directly. If she admits to it, I would ask her to fix the problem or not continue to volunteer.	I would be straight up with my friend and tell her what I heard and ask her to be honest with me. If she is really my good friend then I would have to take her word for it and move on from the situation because there are more important things to be worrying about.	I would take my investigative findings to my friend, the treasurer, and ask where the missing money has gone. I will assure my friend that whatever the case may be, they will still by my friend by the end of this and ask that they tell the truth.	I would ask my close friend to be honest with me and ask if there had been any misuse of funds. Hopefully, I can gain some more insight on the financial events that took place. I would then take my findings and the issue to the executive board of my local fraternity and seek their input.
